# Nonclassical Recrystallization

**DOI:** 10.1002/chem.202002873

**Published:** 2020-10-16

**Authors:** Julian Brunner, Britta Maier, Rose Rosenberg, Sebastian Sturm, Helmut Cölfen, Elena V. Sturm

**Affiliations:** ^1^ Physical Chemistry University of Konstanz Universitätsstraße 10 78457 Konstanz Germany; ^2^ Institute for Solid State Research Leibniz Institute for Solid State and Materials Research Dresden Helmholzstraße 20 01069 Dresden Germany

**Keywords:** analytical ultracentrifugation, mesocrystals, nanocrystals, recrystallization, size separation

## Abstract

Applications in the fields of materials science and nanotechnology increasingly demand monodisperse nanoparticles in size and shape. Up to now, no general purification procedure exists to thoroughly narrow the size and shape distributions of nanoparticles. Here, we show by analytical ultracentrifugation (AUC) as an absolute and quantitative high‐resolution method that multiple recrystallizations of nanocrystals to mesocrystals is a very efficient tool to generate nanocrystals with an excellent and so‐far unsurpassed size‐distribution (PDI_c_=1.0001) and shape. Similar to the crystallization of molecular building blocks, nonclassical recrystallization removes “colloidal” impurities (i.e., nanoparticles, which are different in shape and size from the majority) by assembling them into a mesocrystal. In the case of nanocrystals, this assembly can be size‐ and shape‐selective, since mesocrystals show both long‐range packing ordering and preferable crystallographic orientation of nanocrystals. Besides the generation of highly monodisperse nanoparticles, these findings provide highly relevant insights into the crystallization of mesocrystals.

## Graphical abstract

**Purifying nanocrystals**: The nonclassical recrystallization presents a general purification procedure of nanocrystal dispersions. Analytical ultracentrifugation proves as an absolute high‐resolution method that multiple recrystallizations of nanocrystals to mesocrystals thoroughly narrow size and shape distributions of nanoparticles. Nonclassical recrystallization removes “colloidal” impurities similar to the recrystallization of “classical” (atomic, molecular) crystals.

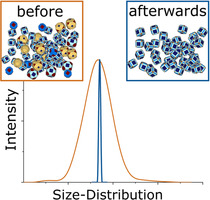

## Introduction

Nanotechnology has been one of the most active fields in materials science in the last decades.^[1]^ Nowadays, the implementation of nanocrystals in various applications is of higher interest than their generation, which is already well established to a certain degree.^[2]^ These applications often demand high quality nanocrystals in size and shape.^[3]^ Nevertheless, direct generation of nanocrystals with a narrow size and shape distribution are still a challenging task.^[4]^ Ordinary synthesis methods often produce nanocrystals with different shapes and high polydispersity indices and no universal purification steps are established. The fractionation of broad nanocrystal size dispersions by ultracentrifugation is a promising and upcoming technique.^[5]^ However, it is not widely used, although it is known from analytical ultracentrifugation (AUC) that nanocrystals can be separated with Ångström size resolution.^[6]^ In contrast to nanocrystals, the chemical synthesis and purification of organic molecular compounds are well investigated.^[7]^

Recrystallization is generally used to significantly increase the purity of chemicals.[^5c^, ^7^] The separation of the crystalline product from “molecular” impurities by multiple recrystallization has been used for many years. Unlike molecular compounds, nanocrystal synthesis additionally produces “colloidal” impurities (i.e., nanoparticles, which are different in shape and size from the majority). Nanocrystals can assemble to mesocrystals by “oriented aggregation”, which is a reversible process and even single crystals by “oriented attachment”, which is not reversible and therefore can be seen as analogy to atoms, ions, or molecules in “classical” crystallization.[^7^, ^8^] Hence, multiple recrystallization of mesocrystals from nanoparticles to remove “molecular” and “colloidal” impurities should be a desirable tool for materials science and nanotechnology if the crystallization principles on the atomic and molecular scale can be transferred to the nanoparticle scale.

The processes for particle‐mediated crystallization, involving nanoparticles (so‐called “nonclassical crystallization”) are so far not well studied.[^3a^, ^4b^, ^8a^, ^9^] In this context, recrystallization is one of the most interesting phenomena. In most studies, the reported “superstructure recrystallization”^[10]^ does not involve the reversible redispersion and reassembly of nanoparticles into a superstructure. In these cases, the term recrystallization is rather used to describe the secondary crystallization of the new crystalline phase from the initial amorphous or crystalline metastable precursor phase, and/or recrystallization in terms of Ostwald ripening or nanoparticles fusion. Therefore, such processes do not allow to achieve dispersions or superstructures of nanoparticles with narrow size and shape distributions, as presented here. Nevertheless, size separation of nanoparticles via crystallization processes is reported for a few systems.^[11]^ The “Zenon‐packing” describes the self‐organization of polydisperse colloidal particles,^[11a]^ meaning that big particles form the core of the assembly (with hyperbolic geometry) and smaller particles are located on the outside areas or excluded. The exclusion of impurity particles was detected in charged colloidal silica crystals with time resolved confocal laser scanning microscopy.^[11c]^ Shape separation of nanoparticles can be enabled by different solubilities of the building block shapes as well as by DNA‐programmable nanoparticles.^[12]^ Additionally, recrystallization and zone‐melting of charged silica particles leads to an improvement of crystal quality and crystal size.^[13]^ A “self‐cleaning effect” demonstrated by microscopy techniques due to “colloidal recrystallization” was reported for metallic nanoparticles by rinsing impurities from the surface of a superstructure,^[14]^ while in case of semiconducting nanoparticles a size‐selective formation was observed.^[15]^ Applicability to chemically different nanocrystals or scientific considerations provided by sufficient analytical examination of the purification process itself are, however, missing for the reported approaches.

## Results and Discussion

**Figure 1:**
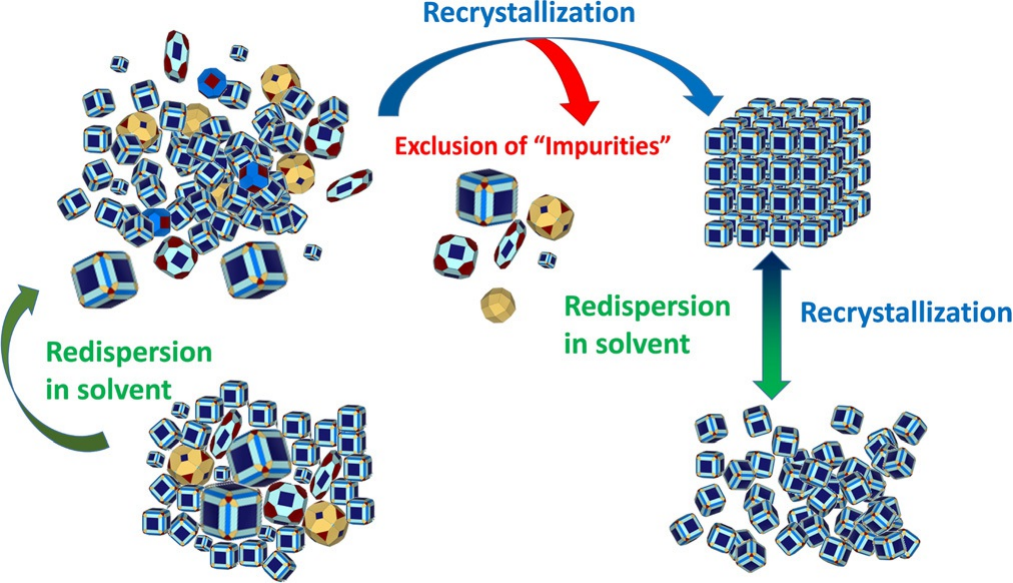
Nonclassical recrystallization. First, the “impure” colloidal agglomerate is redispersed in solvent and afterward recrystallized to mesocrystals. The recrystallization step tends to exclude “colloidal impurities”. The crystallization of mesocrystals is reversible. Therefore, “colloidal impurities” can be removed by repeated recrystallization, separating the supernatant from the mesocrystals. This process accumulates nanocrystals of higher quality within the mesocrystals, which can be again redispersed in any solvent.

Here, we show that nanocrystals with narrow size and shape distribution and high‐quality mesocrystals can be produced by multiple recrystallization of nanocrystals to mesocrystals (Figure 1). Furthermore, the relevance of high‐resolution and statistically relevant quantitative AUC investigations on nanoparticles is nicely illustrated. The nanocrystal as well as mesocrystal quality increases with each crystallization cycle. Even by applying an optimized synthesis procedure, the obtained nanocrystals within one batch can vary in their shape and size as demonstrated by the PSD (particle‐size distribution) and sedimentation coefficient distribution (g(s)). Nanocrystals assemble into lattices meanwhile excluding “colloidal” impurities and narrowing the size and shape distribution of the nanoparticles. Thereby, performing multiple recrystallization steps, nanocrystal batches with the narrowest size‐distribution up to a polydispersity index (PDI_c_, subscript “c” stands for the diffusion broadening correction) of 1.0001 (Batch I, third crystallization) can be obtained. To the best of our knowledge, this is the best value for nanoparticle polydispersity, which was reported so far. Even small changes in size can lead to accumulation of the bigger nanocrystals within the mesocrystals leaving the smaller ones in the supernatant.

**Figure 2:**
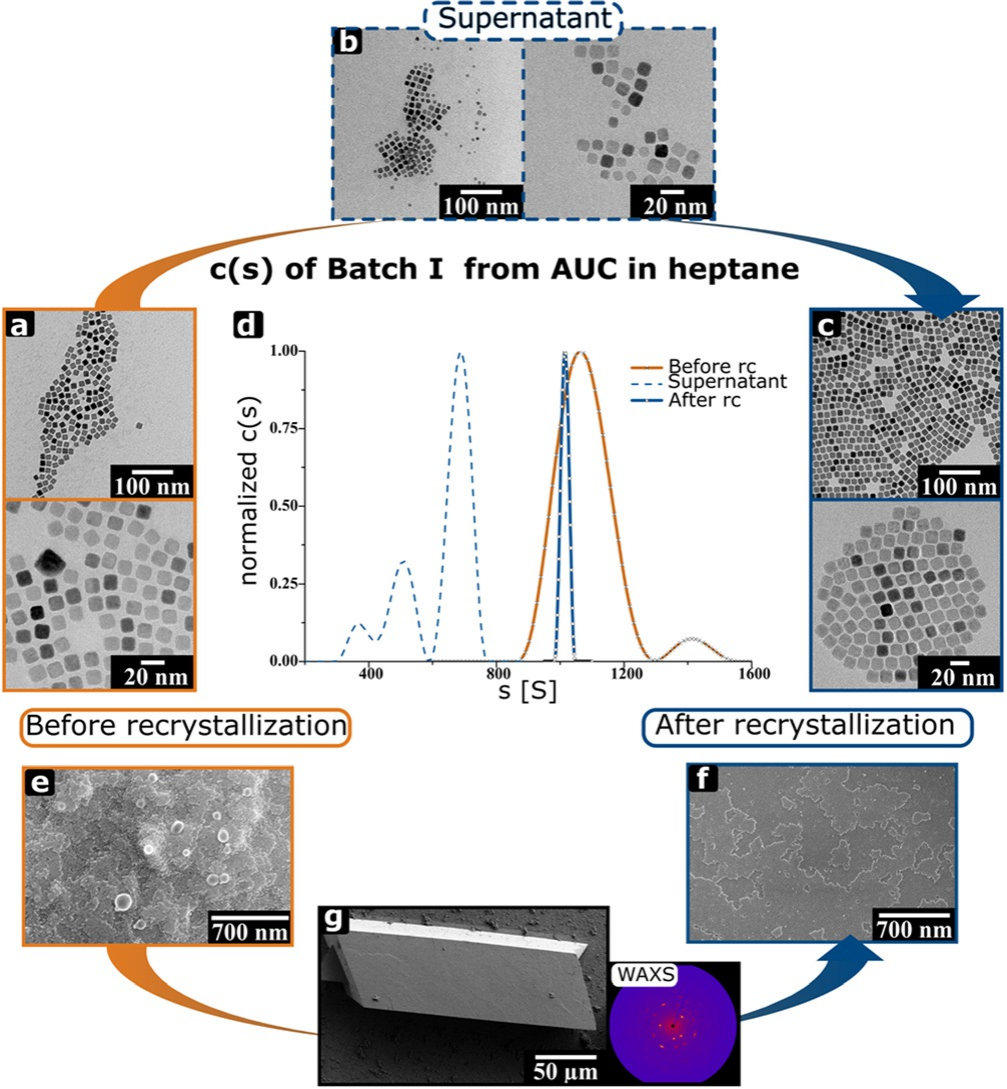
Important steps of “nonclassical” recrystallization. a–c) TEM images at each stage of the nanocrystals (Batch I) before and after the crystallization of mesocrystals and the supernatant. The nanocrystals from the supernatant were not incorporated in the mesocrystal. d) The normalized diffusion‐corrected sedimentation coefficient distribution narrows after a recrystallization (rc) step. The dotted line presents the polydispersity of the supernatant. e, f) The SEM images clearly demonstrate how the quality of the mesocrystal surface increases after the recrystallization step. g) Representation of a rhombohedral shaped mesocrystal Type 1. Inset: texture‐like WAXS pattern.

We performed “nonclassical” recrystallization of iron oxide nanocubes with initially different size‐distributions from various solvents. At first, slightly truncated nanocubes with a hydrodynamic diameter (*d*_H_) between 9–15 nm were synthesized according to the literature.^[16]^ The nanocrystals can be transferred to any suitable solvent after synthesis. In order to generate mesocrystals, the nanocrystal dispersions were destabilized by ethanol via the gas phase (see Figure S1 for light microscopy image).^[17]^ The most important steps of the crystallization processes are analysed by AUC and electron microscopy measurements and shown in Figure 2. The nanocrystal dispersions, a rhombohedral mesocrystal and the mesocrystal surfaces of Batch I are presented before (*d*_H_=15±0.3 nm) and after (*d*_H_=14.7±0.3 nm) the recrystallization (abbreviated “rc” in the figures) from heptane in Figure 2. Additionally, the supernatant has been investigated after the assembly process was finished. The diffusion‐corrected^[18]^ sedimentation coefficient distributions (c(s)) of the nanoparticle dispersion (Figure 2 d) show that the polydispersity of the redispersed nanoparticles from mesocrystals narrows due to the crystallization. The PDI_c_ (diffusion broadening corrected polydispersity index) decreases from 1.0126 to 1.0001. This is also confirmed by transmission electron microscopy (TEM) imaging of the nanocrystal dispersions before and after crystallization as well as of the separated supernatant (Figure 2 a–c, Figure S2). AUC and TEM analyses of the supernatant show that irregular nanocrystals of a broader size distribution are excluded from mesocrystals during the assembly process. AUC measurements indicate different nanocrystal species. It must be noted that the supernatant contains unknown amounts of ethanol from the destabilization process, which significantly lowers the sedimentation coefficient because larger amounts of ethanol increase the viscosity and density of the mixture (see Methods section in the Supporting Information for further information).^[19]^ Hence, c(s) is shifted. Beside the sedimentation coefficient distributions, Figure S3 depicts the colour of the supernatant after several crystallization cycles. With each crystallization cycle, the supernatant becomes clearer indicating that less and less nanocrystals with size/shape mismatches get removed with subsequent recrystallization. The scanning electron microscopy (SEM) images of the mesocrystal surface demonstrate that the “quality” of the mesocrystals increases due to recrystallization and they accumulate building blocks with narrower size and shape distribution (Figure 2 e and f, Figures S4–S8). Figure 2 e–g shows the improvement of packing order of the nanoparticles in the mesocrystal, which is reflected by significant decreasing of the surface roughness and defectiveness. Figure 2 e displays the initial mesocrystal surface before the recrystallization step and Figure 2 f and g after the recrystallization. The imperfect building blocks are excluded by the mesocrystal crystallization process (Figure 2 g) and remain in solution. The inset Figure 2 g shows a texture‐like wide‐angle X‐ray diffraction pattern of such a faceted rhombohedral mesocrystal to demonstrate the orientational order of crystalline building blocks. However, some imperfect nanocrystals remain and get included into the crystal lattice as impurity, which locally distorts the crystal lattice just like it is known from “molecular” impurities in “classical” (atomic, molecular) crystals. This can be seen by comparing Figures S5 and S6.

**Figure 3:**
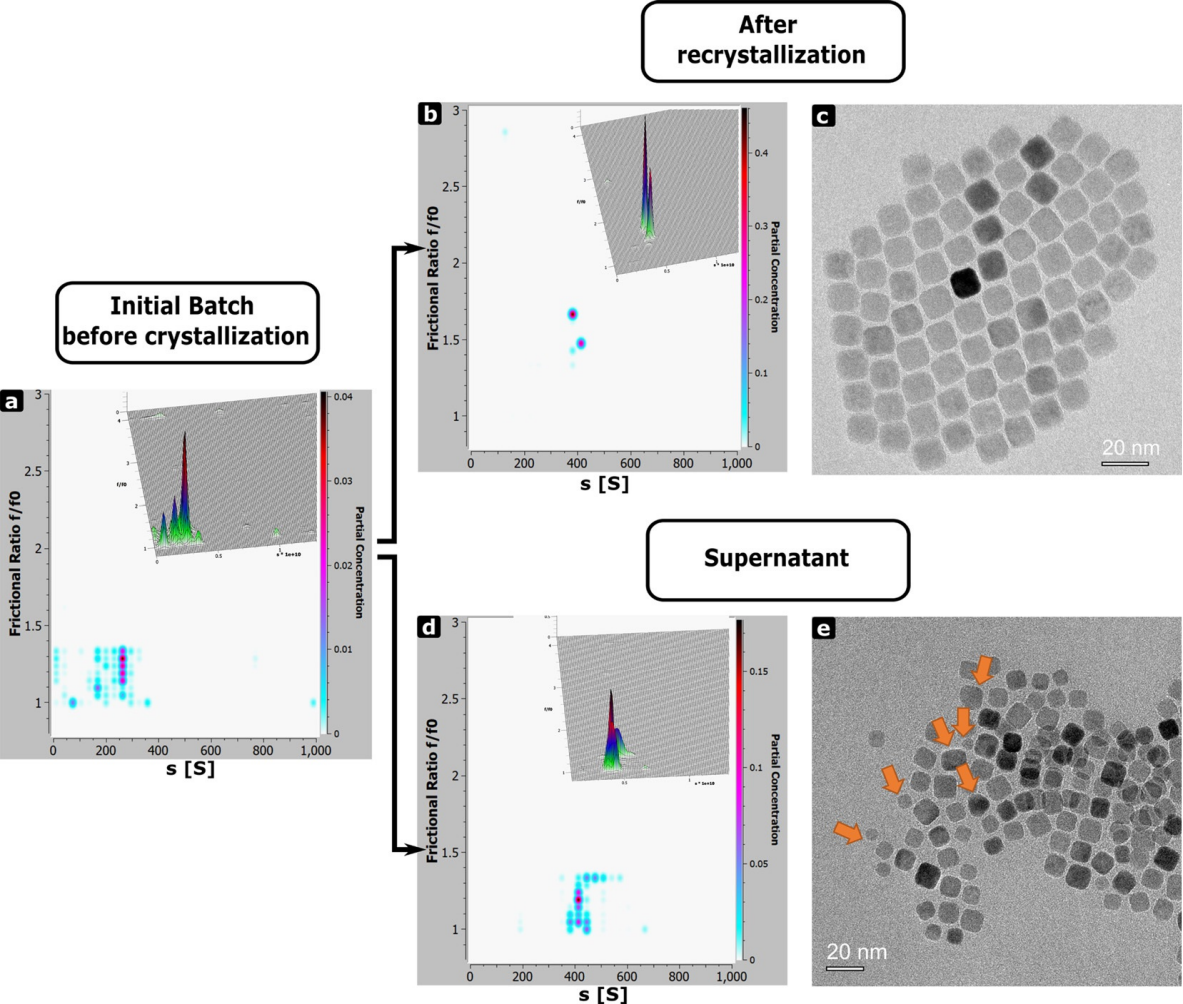
Effect of recrystallization on shape selectivity of nanoparticles. a, b, d) 2D plots obtained by 2 DSA‐MC analysis of AUC data (Figure 2 d) showing the relation of sedimentation coefficient and f/f_0_ for the nanocrystals (batch I) before and after the crystallization of mesocrystals and supernatant. Insets top right show 3D representations of the plots, the colour gradient indicates the partial concentration of the species. c, e) Exemplary TEM images of the nanoparticle batch after the fifth recrystallization cycle and in supernatant. Orange arrows highlight several particles of non‐cubic shape. The analysis of the shape distribution is shown in Figure S8.

Multiple recrystallization is also expected to improve the shape selectivity of nanoparticles due to the fact that in mesocrystal nanoparticles are arranged not only in a long‐rage ordered superlattice, but also show the preferable crystallographic orientation. In order to verify this phenomenon, we performed a detailed evaluation of AUC and TEM data collected on nanoparticle dispersions before and after recrystallization, as well as the supernatant. Figure 3 shows the plots of the distribution of the frictional coefficient ratio f/f_0_ vs. the sedimentation coefficient distribution obtained by 2DSA‐MC analysis^[20]^ of AUC data for Batch I, shown in Figure 2 d. The magnitude of the frictional ratios f/f_0_ is reflecting the deviation of nanoparticle shape form the ideal sphere (f/f_0_=1) and also the structure of the solvation shell.[^20^, ^21^] It is obvious that the initial dispersion contains several populations of nanoparticles with slightly different shapes (Figure 3 a). After several recrystallization cycles the shape distribution of the nanoparticles is significantly improved (Figure 3 b), while the population with diverse shape remains in the supernatant (Figure 3 d). These findings are also consistent with the results of TEM analysis of nanoparticle shape distributions (Figures 2 a–c and 3 c,e, Figure S8), which, however, gives not such a good statistics in comparison to AUC.

**Figure 4:**
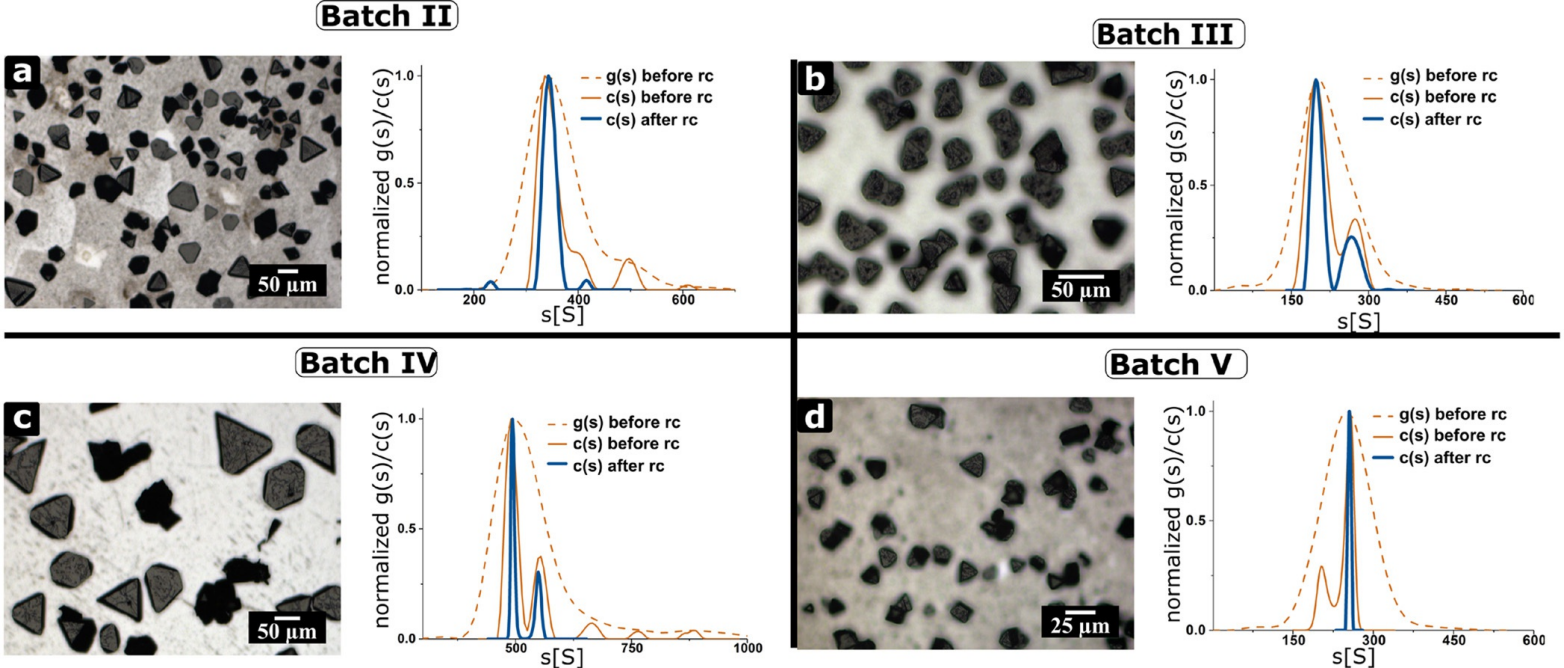
Presentation of other investigated nanocrystal batches and their corresponding mesocrystals from cyclohexane. a–d) Sedimentation coefficient distribution of different nanoparticle batches from cyclohexane before and after a purification step. Batch II–IV contain a majority of larger over smaller “colloidal” impurities, while Batch V contains smaller than larger “colloidal” impurities. Mesocrystals could be obtained for all different nanocrystal batches. Highly monodisperse nanocrystals are received after purification. Please note that the non‐diffusion corrected g(s) envelopes the high‐resolution diffusion‐corrected c(s).

**Figure 5:**
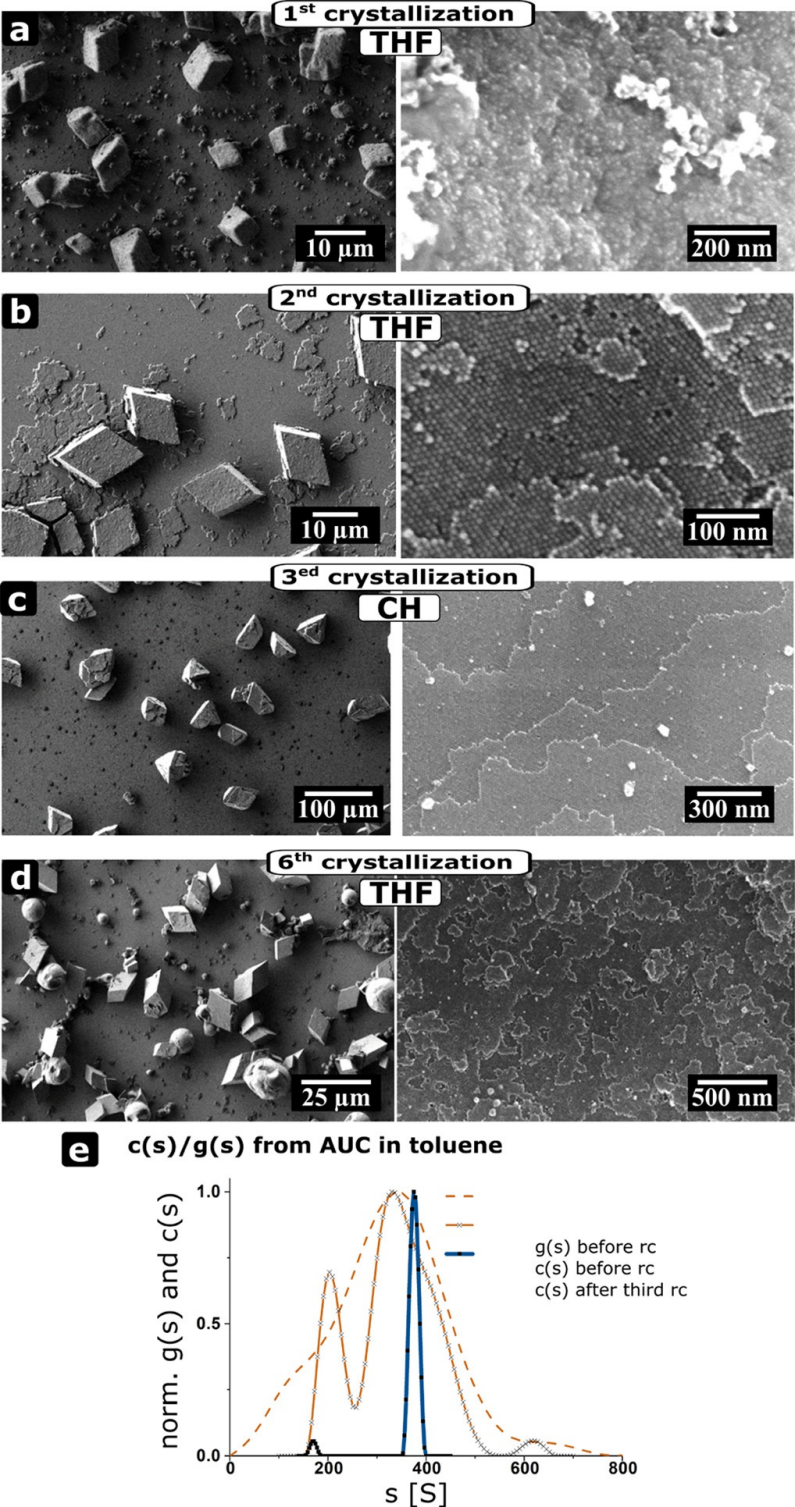
Reversible formation of mesocrystals with different symmetry of the superstructure and the size‐selective recrystallization. SEM images of mesocrystals and their corresponding images after several recrystallization cycles from Batch V. a) After first crystallization in THF b) After the second crystallization in THF c) After the third crystallization in cyclohexane (CH) and d) After the sixth crystallization in THF. e) Normalized c(s) and g(s) before and after three recrystallization cycles.

**Figure 6:**
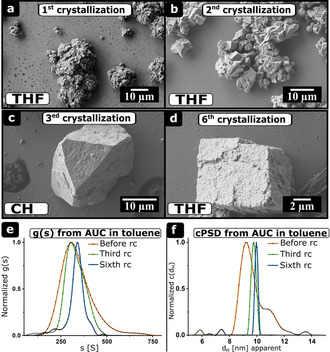
Evolution of mesocrystals from agglomerates. Recrystallization of colloidal agglomerates and mesocrystals from nanocrystal batch III with initially broader PSD and lower quality using THF and cyclohexane (CH). a–d) SEM images of the agglomerates and mesocrystals obtained after several recrystallization cycles. a) First crystallization, b) second crystallization, c) third crystallization, d) sixth crystallization. e, f) g(s) and cPSD from AUC in toluene before the crystallization and after the third and sixth crystallization.

The polydispersity of the nanocrystals decreases due to recrystallization for all investigated nanocrystal batches. The investigated nanocrystal batches were crystallized to mesocrystals from cyclohexane (Figure 4). All nanocrystal batches exhibit mesocrystals with a morphology of a trigonal truncated pyramid. Figure 5 and Figure 6 provide SEM images of the mesocrystal and facet surface for Batch III and V. Figure 4 a–d show the ordinary (dashed line) and diffusion‐corrected (solid line) sedimentation coefficient distribution of the nanocrystal batches. g(s) as the enveloping distribution represents only one species, whereas c(s) shows that different species in the nanocrystal dispersion can be present. In all cases, the distribution before purification (orange) is broader than afterwards (blue), so that the sedimentation coefficient distribution (g(s) and c(s)) of all nanocrystal batches narrows after one or several recrystallization cycles (PDI_c_ for Batch II: 1.0045, Batch III: 1.0229, Batch IV: 1.0026 and Batch V: 1.0001). The recrystallization cycles can remove larger (Batches II—IV in Figure 3) and smaller “colloidal” impurities (Batch V in Figure 4). On the one hand, a size selective nanocrystal assembly removes the other species for nanocrystal Batch I, II and V (Figure 2 d, Figure 4 a and d). Recrystallization removes larger (Figure 4 a) and smaller (Figure 4 d) impurities. These findings are also verified by high‐resolution (HR)TEM images of nanoparticles shown in Figure S7. On the other hand, the purification can lead to a more distinct fractionation of the nanocrystal dispersion (Figure 4 b and c). This distinct fractionation of different nanocrystal species might indicate a simultaneous crystallization of different independent mesocrystal species.

Investigations on nanoparticle Batches III and V revealed highly interesting phenomena. Mesocrystals with different symmetry of superstructure (e.g. analogous to “structural polymorphism”) can be reversibly formed in solvents with different polarity and size and shape fractionation processes occur.^[21a]^ Thus, nanocrystal batch V was transferred to the dispersion agent tetrahydrofuran (THF) (Figure 5). Mesocrystals crystallized from THF exhibit rhombohedral shape similar to Batch I, instead of trigonal truncated pyramidal mesocrystals assembled from cyclohexane as presented in Figure 4. The experiment with the first crystallization leads to mesocrystals with smooth edges and a disordered surface (Figure 5 a). The quality of these mesocrystals increases drastically after two crystallization cycles (Figure 5 b). The edges of the mesocrystals are sharp and the surface is more ordered and less rough than after the first crystallization. The crystallization of the nanocrystals led again to mesocrystals with a trigonal truncated pyramidal morphology (Figure 5 c) when the dispersion has been transferred to cyclohexane. Finally, the last crystallization in THF, led to morphologies with a rhombohedral morphology (Figure 5 d). The normalized c(s) and selected g(s) of the purification are given in Figure 5 e. Before the first crystallization, the g(s) (*d*_H_=9.8±0.2 nm), forms a shoulder around 6 nm (dashed orange line). The shoulder indicates a second nanocrystal species, which is confirmed by the c(s) (orange line, PDI_c_=1.0709). The different species completely vanishes for the experiment after several recrystallization cycles (blue line, *d*_H_=10.4±0.2 nm, PDI_c_=1.0122) and highly monodisperse nanocrystals are obtained. This observation reveals that the “nonclassical” recrystallization can also be a size and shape‐fractionating process to increase nanocrystal and mesocrystal quality and remove “molecular” and “colloidal” impurities. Moreover, it shows that the morphology (e.g. symmetry of the superlattice) of mesocrystals can be reversibly changed.

Similar experiments on nanocrystal batch III demonstrate the importance of the recrystallization steps for the successful formation of mesocrystals (Figure 6). For nanocrystal batch III, mesocrystals could be only obtained after several recrystallization steps. The first two SEM images show disordered agglomerates (Figure 6 a and b). After the second recrystallization, some agglomerates unveil ordered regions. Not until the third recrystallization cycle, small mesocrystals had been observed (Figure 6 c and d).

The change of morphology due to the change of dispersion agents once again demonstrates the reversibility of the “nonclassical” recrystallization and the easy polymorph control. The g(s) and the diffusion broadening corrected particle size distribution (cPSD) narrows drastically for several recrystallization cycles (Figure 6 e and f). The PDI_c_ and the calculated nanocrystal diameter (*d*_H_=9.2±0.2 nm, PDI_c_=1.0487) differs significantly from the third (*d*_H_=9.8±0.2 nm, PDI_c_=1.0487) and the sixth (*d*_H_=10±0.2 nm, PDI_c_=1.0005) recrystallization step. The significant narrowing of the distributions shows two important facts. On the one hand, narrow size and shape distributions of the building blocks are important to form high quality ordered superstructures (i.e. mesocrystals, see Batch I). On the other hand, this is not the only guiding principle for the formation of perfect mesocrystals. If one compares the PDI_c_ and mesocrystals of this purified nanocrystal batch with nanocrystal batches of higher quality without purification (batches I, II, IV), the obtained mesocrystals are of poor quality. We note a shift of the main cPSD and g(s) peaks in Figure 5 and 6. The main peak shifts from *d*_H_=9.2 nm to *d*_H_=10.0±0.2 nm in case of nanocrystal Batch III and in case of Batch V from *d*_H_=9.8 nm to *d*_H_=10.4±0.2 nm. We observed such a shift to bigger values for asymmetric cPSD and g(s) curves, which is reasonable. Smaller nanocrystals should preferably stay within the supernatant, since they exhibit weaker attractive van der Waals forces than larger nanocrystals.[^15a^, ^15c^] Hence, larger nanocrystals tend to aggregate before the smaller nanocrystals. In addition, the smaller the nanocrystals, the slower the sedimentation should proceed. Smaller nanocrystals of worse quality should also be excluded thoroughly.

## Conclusions

In summary, we reported the purification, shape‐ and size‐narrowing and fractionating effects of recrystallization of iron‐oxide nanocubes to mesocrystals due to their reversibility in formation similar to “classical” recrystallization of molecular compounds. This work extends the knowledge of crystallization processes and nanocrystals as building blocks. It also demonstrates that AUC is a fundamental and universal high‐resolution tool for research in nanoscience. We analysed the mesocrystals, the redispersed particles from mesocrystals and the supernatant based on AUC analyses and electron microscopy techniques. The analysis shows quantitatively that the PDI_c_ of the nanocrystal batch decreases with each recrystallization cycle and size distributions of particles with PDI's up to 1.0001 are accessible. Due to the fact that AUC counts each nanoparticle in the dispersions, the PDI_c_ calculations presented here are statistically highly relevant. “Molecular” and “colloidal” impurities remain within the supernatant similar to “classical” recrystallization. The exclusion of these impurities improves the “quality” of the mesocrystal (by showing less defects, clear surfaces and faceted shape) dramatically immediately after the first recrystallization. Even nanocrystal batches with an initially broad size distribution form mesocrystals after several recrystallization cycles while before that, only disordered agglomerate formation is observed. We also demonstrate the reversible formation of mesocrystals with a different symmetry of the superstructure (e.g. analogues to “structural polymorphism”) from the same nanocrystals by a simple variation of the solvent. All in all, our findings give fundamental insights into the crystallization processes of mesocrystals from nanoparticles, show the analogy of “nonclassical” crystallization to “classical” crystallization and give an important tool to obtain monodisperse nanoparticles after synthesis to construct highly ordered superstructures (i.e. mesocrystals), which cannot be obtained by approaches without further purification.

## Experimental Section

### Methods

Nanocrystals were synthesized as described in the literature.^[16]^ Mesocrystals were crystallized via gas phase diffusion destabilization in different solvents (cyclohexane Uvasol 99.9 %; THF VWR Chemicals 99.9 %; toluene VWR Chemicals 99.9 %; ethanol VWR Chemicals 99.9 %).^[17]^ A nanocrystal dispersion with 3 μL mL^−1^ oleic acid (Sigma–Aldrich 99.9 %) was destabilized with 1:1 ratio (ethanol : solvent). The recrystallization process involves that the coloured supernatant is removed via syringe subsequent the crystallization process is finished (see Figure S2). Afterwards fresh solvent is injected and the mesocrystals are redispersed using a (fresh) syringe. The glass vial containing the nanocrystal dispersion also contained a polished silicon snippet (Siegert Wafer, Germany) for analyses via SEM (Zeiss CrossBeam 1540XB). The silicon snippet had been washed with toluene prior to crystallization. Crystals were collected and redispersed for AUC measurements. Small traces of nanocrystal dispersion were investigated with an AUC (Optima XL I, Beckman Coulter) and TEM (Zeiss Libra120 TEM microscope operating at 120 kV) and (HR)TEM (Titan^3^ TEM microscope operating at 300 kV) after purification. The used TEM grids (2 nm Carbonfoil) were purchased from Quantifoil. TEM images were analysed with DigitalMicrograph ® Gatan Microscopy Suite 3 software (Gatan Inc., ver. 3.41.2938.1). The PDI_c_ ^[22]^ and the c(s) using the Tikhonov‐Philips second derivative regularization had been calculated according to literature using the program SEDFIT version 16.1c (https://sedfitsedphat.nibib.nih.gov/software/default.aspx). UltraScan3^[20b]^ (Version 4.0, revision 5807) was used for performing the two‐dimensional spectrum analysis (2DSA)^[20a]^ The 2DSA‐Monte Carlo (MC) analyses were performed with 50 iterations. g(s) is a model free calculated sedimentation coefficient distribution while c(s) corrects for diffusion broadening. Therefore, a g(s) distribution envelopes the c(s) distribution. For the calculation of the polydispersity, c(s) was applied because it reflects the particle size distribution. The calculation of the particle size, from the sedimentation coefficient distribution is explained in the supplementary information.

## Conflict of interest

The authors declare no conflict of interest.

## Supporting information

As a service to our authors and readers, this journal provides supporting information supplied by the authors. Such materials are peer reviewed and may be re‐organized for online delivery, but are not copy‐edited or typeset. Technical support issues arising from supporting information (other than missing files) should be addressed to the authors.

Supplementary
