# Supplementary material for: Nonclassical Recrystallization
Source: Chemistry. 2020 Oct 16;26(66):15242–8. doi: 10.1002/chem.202002873 (PMC7756702; doi:10.1002/chem.202002873)
Supplement: Supplementary file 1 — Supplementary [file CHEM-26-15242-s001.pdf]

Chemistry–A European Journal

Supporting Information

Nonclassical Recrystallization

Julian Brunner,[a] Britta Maier,[a] Rose Rosenberg,[a] Sebastian Sturm,[b] Helmut Cölfen,*[a] and Elena V. Sturm*[a]

Supporting information

1.1. Supporting information - Methods

The sedimentation coefficient s (unit S = 10^{-13}s) allows the determination of the particle size (d_H) by taking into account that the nanocrystal cube resembles a sphere according to the Stokes-Einstein equation

$$d_H = \sqrt{\frac{18\eta s}{\rho_p - \rho_s}}$$

Thereby, d_H equals the hydrodynamic nanoparticle diameter (the diameter of the hydrodynamically equivalent sphere), s equals the sedimentation coefficient, η the solvent viscosity, ρ_p is the particle density, and ρ_s the density of the solvent. According to literature, the density of the nanocrystal, including their oleic acid shell is 4.299 g/mL.[1]

The sedimentation coefficient for the supernatant (heptane : ethanol) decreases for the same size of the nanocrystals because the density and the viscosity of the solvent mixture increase for larger amounts of ethanol.[2]

1.2. Supporting information - Figures

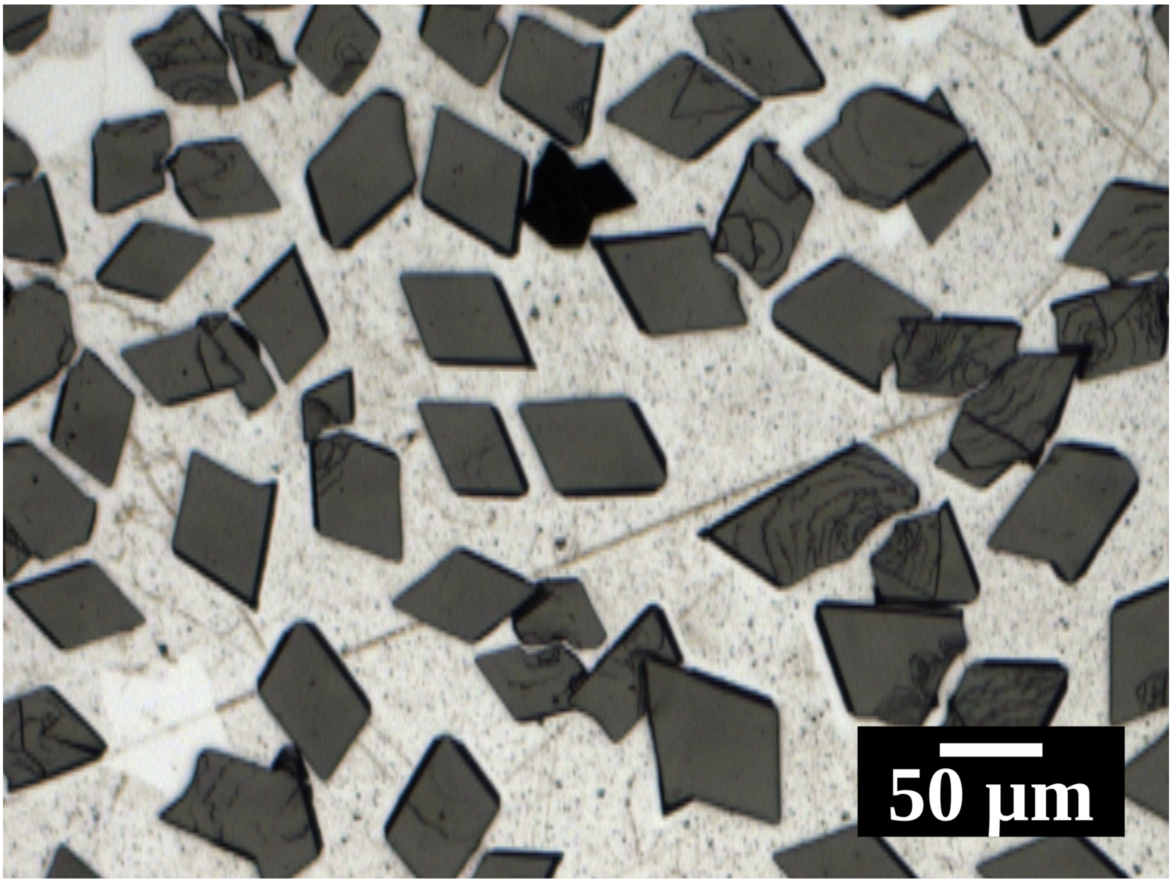

Supplementary Figure S1| Light microscope image of as grown mesocrystals. This light microscope image shows ordinary grown mesocrystals from batch I using heptane as dispersion agent.

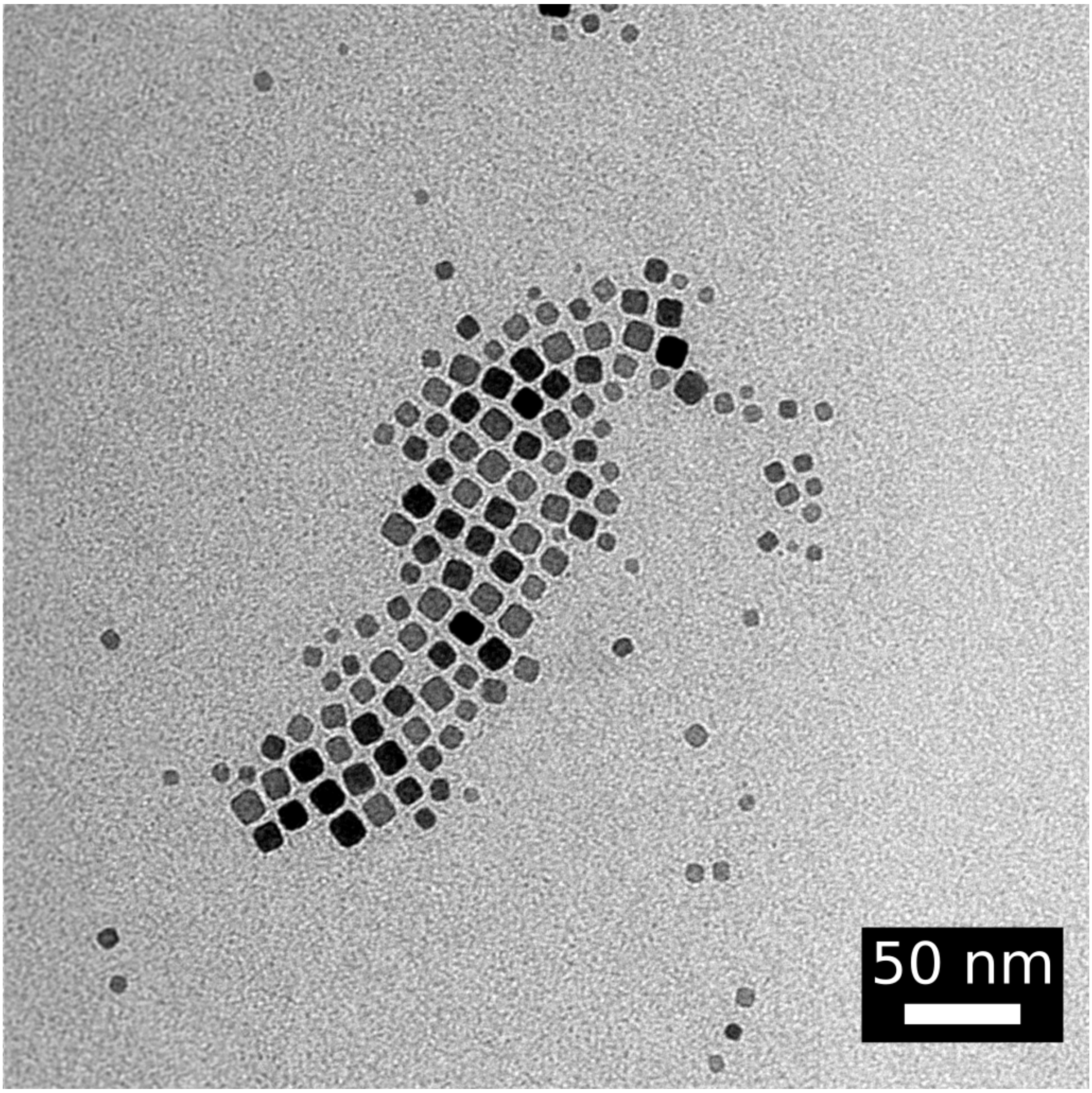

Supplementary Figure S2| TEM image of the supernatant after mesocrystal formation. This TEM image depicts the broad distribution in size and shape of the nanocrystals (batch I) within the supernatant.

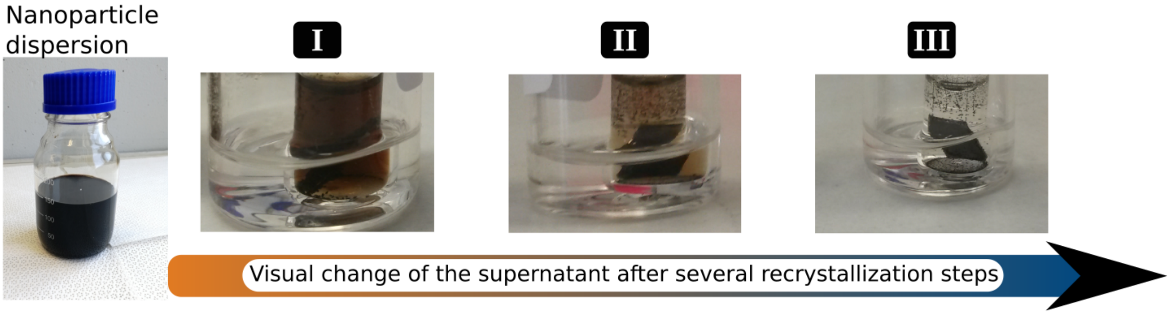

Supplementary Figure S3| The color of supernatant changes after multiple recrystallizations. This figure depicts the colour of the pure nanocrystal dispersion (left) and the change of colour after the nanocrystals self-assemble to mesocrystals. The colour clearly changes for each crystallization step (I-III). Grown mesocrystals can be seen at the edge of the inner glass vials.

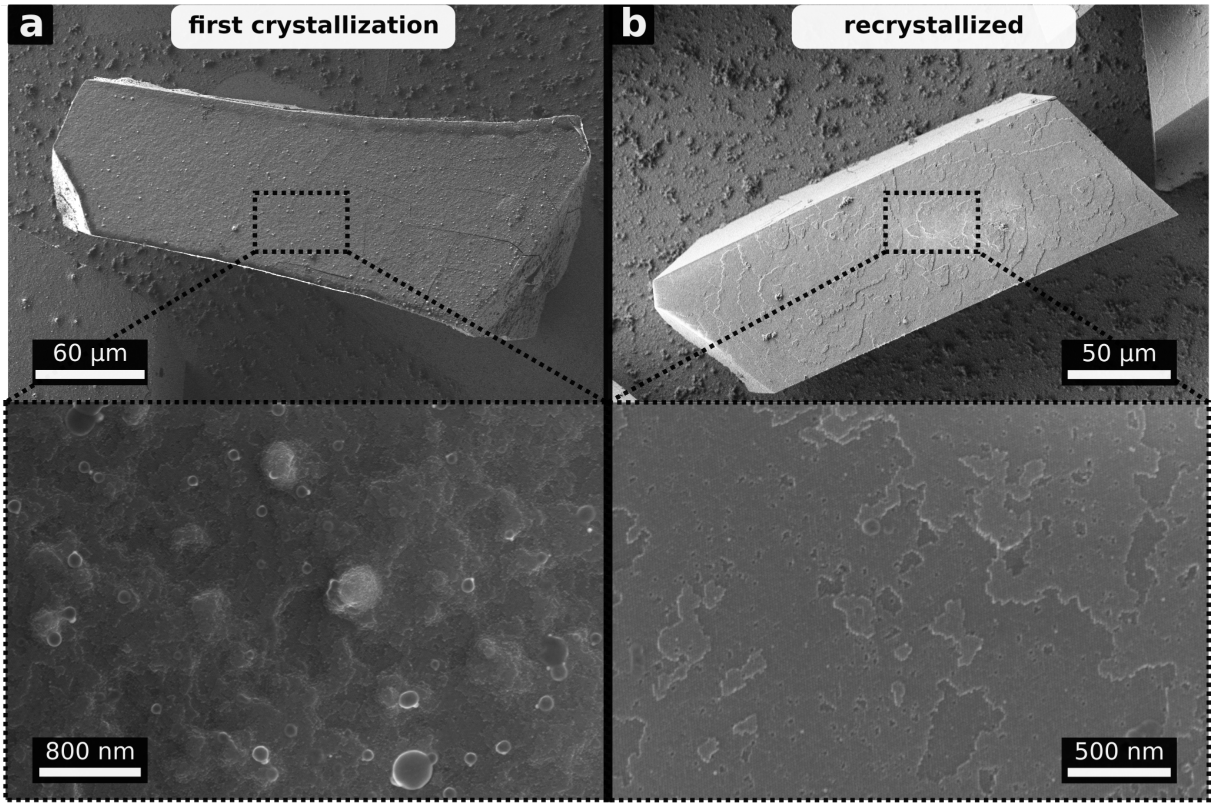

Supplementary Figure S4| Mesocrystal quality improvement. These SEM images depict the improvement of mesocrystal quality before (a) and after (b) several recrystallization steps.

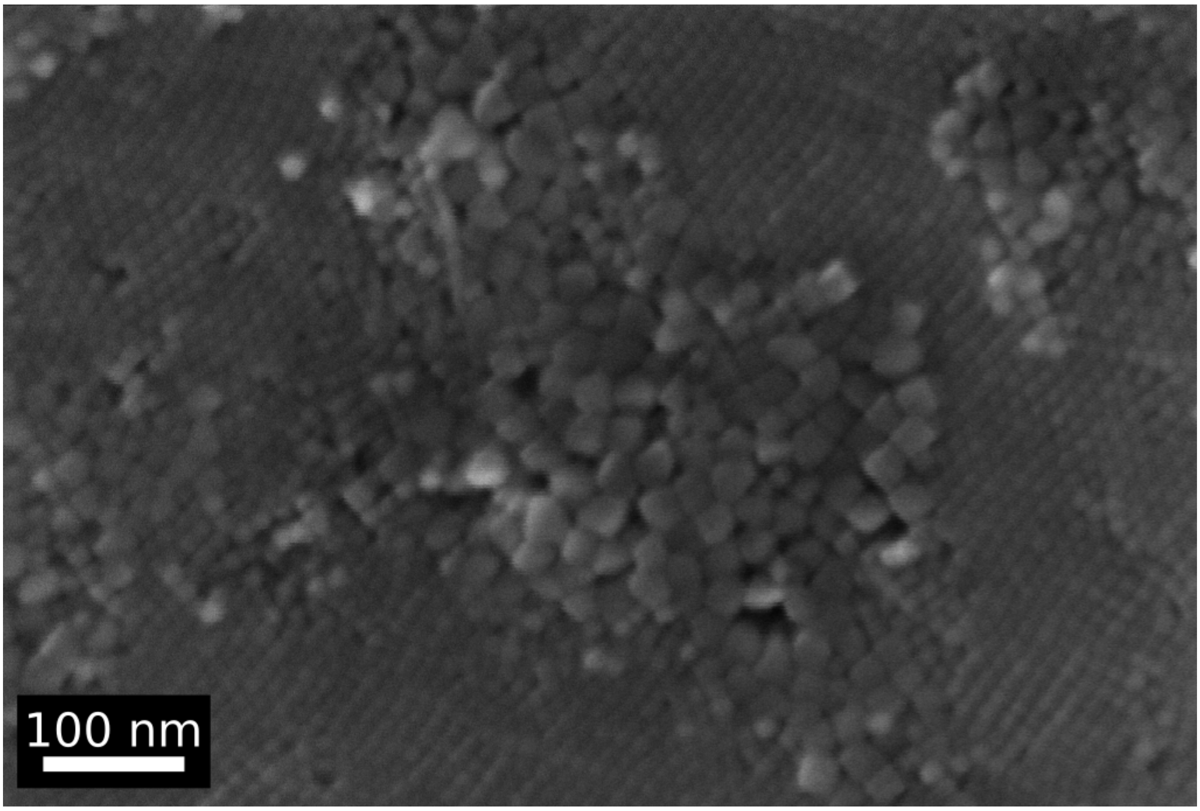

Supplementary Figure S5| Distortions of the mesocrystal superlattice. This SEM image demonstrates distortions in the superlattice of mesocrystals produced by imperfect building blocks as “colloidal” impurities.

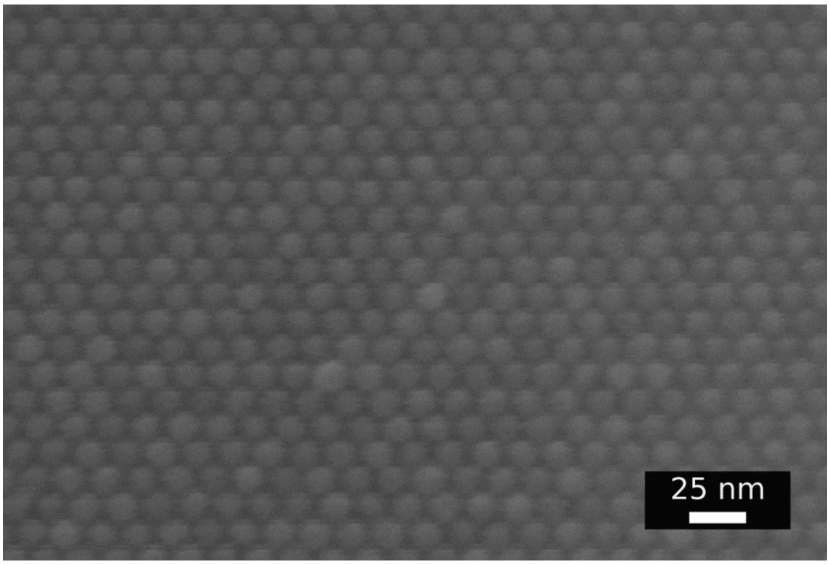

Supplementary Figure S6| Highly ordered packing for recrystallized mesocrystal surfaces. This SEM image demonstrates an almost perfect packing for a recrystallized mesocrystal surface.

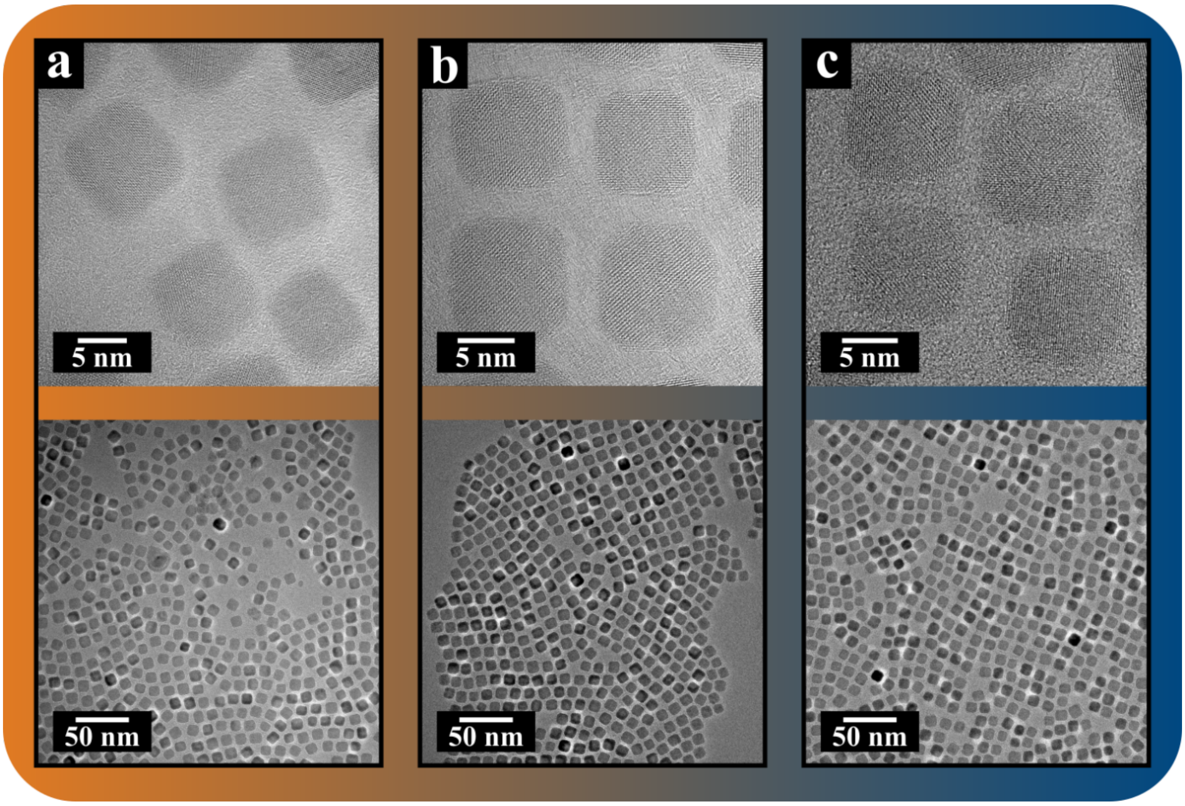

Supplementary Figure S7| (HR)TEM images of nanocrystal batch V. a The nanocrystal dispersion before the purification is polydisperse and contains mishapened nanocrystals. **b** The nanocrystal quality improves after the first purification step. **c** The nanocrystal becomes better furher on - after five purification steps.

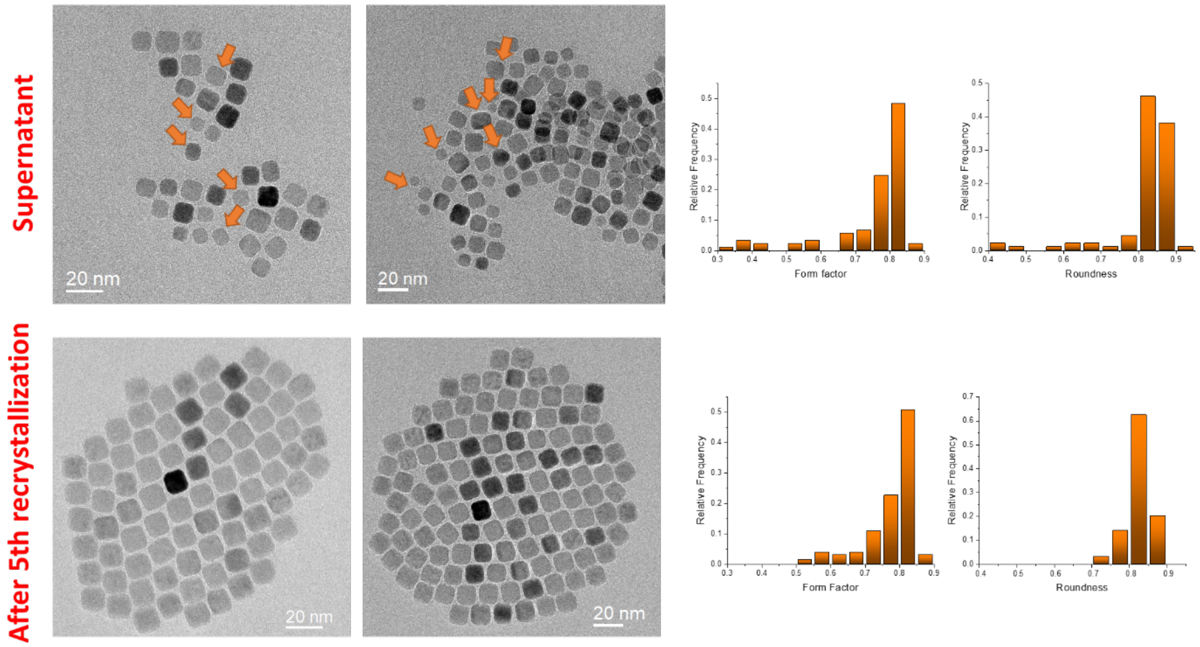

Supplementary Figure S8| TEM images and calculated distribution of form factor ($\frac{4\pi A}{P^2}$) and roundness ($\frac{4A}{\pi L^2}$, where A-area, P- perimeter, L – length) of nanocrystal batch I from supernatant after 1st crystallization (upper row) and batch after 5th recrystallization (bottom row).

References

[1] J. Brunner, I. A. Baburin, S. Sturm, K. Kvashnina, A. Rossberg, T. Pietsch, S. Andreev, E. Sturm (née Rosseeva), H. Cölfen, *Adv. Mater. Interfaces* **2017**, *4*, 1600431.

[2] S. Kouris, C. Panayiotou, *J. Chem. Eng. Data* **1989**, *34*, 200–203.
